# Sporotrichosis: an overview of the neglected disease reported worldwide in the last five years

**DOI:** 10.1590/0037-8682-0211-2025

**Published:** 2025-12-19

**Authors:** João Victor Moura Rosa, Juarez Antônio Simões Quaresma

**Affiliations:** 1 Universidade do Estado do Pará, Programa de Pós-Graduação em Biologia Parasitária na Amazônia, Belém, PA, Brasil.

**Keywords:** Sporotrichosis, *Sporothrix* spp, Neglected zoonosis, One health

## Abstract

Sporotrichosis is a fungal infection caused by species belonging to the genus *Sporothrix*. It primarily affects domestic cats, which are considered definitive hosts, and can be transmitted to humans and other animals in various regions worldwide. We compiled information on the clinical aspects, diagnoses, clinical manifestations, prevention, and therapeutic management of cases reported in the literature between 2018 and 2022. The databases used were BVS-Bireme, PubMed, Scopus, and Web of Science. In total, 121 studies were included. *S. brasiliensis* emerged as the most prevalent species, although *S. schenckii* and *S. globosa* were also reported. This analysis highlights the global increase in zoonotic sporotrichosis and reinforces the importance of collaborative public health strategies based on the One-Health concept.

## INTRODUCTION

Sporotrichosis is a granulomatous subcutaneous mycosis caused by fungi of the genus *Sporothrix* that can infect humans and a variety of animal hosts^1^. Four species have accounted for most clinical cases in recent decades: *S. schenckii* sensu stricto, *S. globosa*, *S. brasiliensis,* and *S. luriei* ^2^ ^-^ ^4^.

*Sporothrix schenckii*, the first species to be described, predominates in the Americas, South Africa, and Australia^5^. In Asia, particularly China, *S. globosa* is the most frequently isolated species. *Sporothrix brasiliensis* circulates across South America^6^, with an especially high incidence in Brazil^7^, and has recently been detected in the United Kingdom^8^. Although the European records remain sparse, infections caused by *S. schenckii*, *S. globosa,* and *S. mexicana* have been confirmed^9^. Close contact between humans and animal hosts, particularly domestic cats, facilitates zoonotic transmission and underscores the global significance of this disease^6^ ^,^ ^9^.

Over the past five decades, sporotrichosis has emerged as an escalating public health challenge, driven by rising case numbers, the appearance of antifungal-resistant strains, and the geographic expansion of outbreaks^9^ ^-^ ^10^.

In this context, our study analyzed publications from 2018 to 2022 to map the worldwide distribution of sporotrichosis in humans and other animals, thereby enhancing the epidemiological understanding and informing future research directions.

## METHODS

This systematic review was conducted in accordance with the Preferred Reporting Items for Systematic Reviews and Meta-Analyses (PRISMA)^11^.

### Search Strategy and Study Selection

Potentially relevant studies were searched independently between April and May 2023 using the following databases: the Virtual Health Library (BVS; bvsalud.org/), PubMed Central (pubmed.ncbi.nlm.nih.gov/), Scopus (scopus.com), and Web of Science (webofknowledge.com/). The descriptors used included: “Sporotrichosis” AND “one health” AND “environmental impacts”; for searches in English, Spanish, and Portuguese.

Duplicate files retrieved through electronic searches were excluded from the databases to select studies for full-text reading. The titles and abstracts of the remaining files were screened to select those to be read in full in the next stage. The full texts of all selected studies (electronic search and reference screening) were examined according to the eligibility criteria (Table 1).

**TABLE 1:** Eligibility criteria adopted for the selection of studies.

Continent/Country	Species	Nº of case reports	Vulnerable population	Lesion type	Diagnostic	Treatment	Reference
**North America**							
Canada	*S. schenckii* complex	1	Male	CF	B	ITZ (200 mg/day)	11
United States	*S. schenckii*	9	Male (7)	CD; EX	CM	ITZ (200 mg 1-2x/day)	12-20
	*Sporothrix sp.*		Female (2)		PCR	PSZ (300mg/2x/day)	
Mexico	*S. schenckii* complex	120	Male (39)	CD; CF; LC	C; PCR; B; CM	ITZ (200 mg/day)	21-26
	*S. schenckii*		Female (43)			IK (4-6 ml/3x/day)	
	*Sporothrix sp.*		N. I. (39)			C	
**Central America**							
Panama	*S. schenckii* complex	1	Male	CF	CM	ITZ (100 mg/day)	27
**South America**							
Argentina	*S. schenckii* complex	1	Female	LC	B; CM	ITZ (10 mg/kg/day)	28
Bolivia	*S. schenckii*	1	Female	CF	B; EMD	IK	29
Brazil	*S. schenckii* complex	7.946	Male (836)	C; B; BAL; E; ELISA; EMD; MALDI-TOF MS; N; CM; PCR	LC; CF; CD; EX; NC; S	AmB intravenous 4 mg/kg/day; FLZ; KTZ; ITZ (100-400 mg/day); IK (2,1 g/day); PRD (40 mg/day) (800 mg/day); TBF (500 mg/day); C; Thermotherapy (40-45 ºC, 3x/day 20 min); Lipossomal AmB (150 mg/kg)	8,30-91
	*Sporothrix sp.*		Female (754)				
	*S. brasiliensis*		Canine (407)				
	*S. schenckii*		Feline (3.701)				
	*S. schenckii* complex		N. I. (6.356)				
Colombia	*S. schenckii*	10	Male	LC	EMD; CM	N.I.	92
Equador	*Sporothrix sp.*	1	Feline	CF	C; CM	ITZ (100 mg/day); Baths with chlorhexidine and miconazole shampoo	92
Paraguai	*S. brasiliensis*	106	Male (11)	LC; CD; CF	EMD; CM; PCR	ITZ (200mg/day); IK (5 gotas/day); AmB (1 g/day)	93-96
	*S. globosa*		Female (95)				
	*Sporothrix sp.*		N.I. (6)				
	*S. schenckii*						
Peru	*S. schenckii*	6	N. I.	N.I.	CM; PCR	N.I.	97
Uruguai	*Sporothrix sp.*	157	Male (152)	LC; CF	EMD	BAN; ITZ	98
			Female (5)				
**Asia**							
China	*Sporothrix sp.*	5.679	Male (2.069)	LC; CD; CF; D; EX;	C; CM; PCR;	IK (10% 0.5-1 g/day);	99-106
	*S. globosa*		Female (3.610)			ITZ (6 mg/kg/day);	
						TBF (5 mg/kg/day)	
South Korea	*S. globosa*	10	Male (2)	CF	CF	ITZ (100 mg/day); TBF	107-108
			N.I. (8)				
India	*S. schenckii* complex	67	Male (24)	LC; CF	B; C; CM; PCR	ITZ (100 mg/2x/day);	109-111
			Female (24)			TBF (250, 500 mg/day)	
			N.I. (19)				
Malaysia	*S. schenckii*	4	Male (2)	CD; CF; EX	B; CM; PCR	ITZ (200 mg/2x/day);	112-115
			Female (2)			FCZ (eye drops 2/2 h);	
						AmB (5 mg/single dose)	
Thailand	*S. schenckii*	2	Female (1) Feline (1)	CF; EX	B; C; N; CM; PCR	Terramycin (oxytetracycline hydrochloride with polymyxin B sulfate) 4x/day; ITZ (10 mg/day)	116-117
**Africa**							
Madagascar	*S. schenckii*	3	Male (2)	LC;	B; CM; PCR; MALDI-TOF MS	PSZ; ITZ; AmB; TBF;	118-121
			Female (1)	EX		Isavuconazonium	
South Africa	*S. schenckii*	171	Male (125)	CF		ITZ (200 mg/day)	
			Female (46)	CD			
**Europe**							
United Kingdom	*S. brasiliensis*	6	Female (3)	CF	B; CM; PCR	ITZ (200 mg/day)	1, 122-123
	*S. humicola*		Feline (1)			ITZ (10 mg/kg PO q24h)	
Germany			Quoll (2)				
**Oceania**							
Australia	*S. chilensis*	2	Feline (1)	N.I.	CM	ITZ (10 mg/kg);	124-125
	*Sporothrix sp.*		Male (1)		PCR	PSZ (7.5 mg/kg/day)	
						SUBA-ITZ; (Lozanoc)	

**Legend: B:** Biopsy; **CM:** Mycological culture; **C:** Cytology; **EMD:** Direct mycological examination; **N:** Necropsy; **MALDI-TOF MS:** Matrix assisted laser desorption time of flight mass spectrometry; **ELISA:** Enzyme-linked immunosorbent assay; **PCR:** Polymerase chain reaction; **BAL:** Sputum/bronchoalveolar lavage; **EPS:** Serum protein electrophoresis; **LC:** Lymphocutaneous; **CF:** Fixed cutaneous; **S:** Systemic; **EX:** Extracutaneous; **CD:** Disseminated cutaneous; **Inc:** Inconclusive; **NC:** Unclassified; **N.I.:** Not reported; **ITZ:** Itraconazole; **AmB:** Amphotericin B; **PSZ:** Posoconazole; **IK:** Potassium Iodide; **C:** Cryosurgery; **FLZ:** Fluconazole; **KTZ:** Cetoconazole; **PRD:** Prednisone; **TBF:** Terbinafine; **BAN:** Cephradine; **SUBA ITZ:** Super BioAvailability itraconazole;

### Data Extraction and Summary of Results

Relevant data were extracted, prioritizing the following items: study identification (year and surname of the first/other author(s)), study title, nature of the study (epidemiological study, clinical case reports), study location, sample (number of cases, animal species, patient gender, age), clinical aspects (diagnosis, form, species, and treatment), and other relevant information. Data were analyzed using Microsoft Excel® spreadsheet. The studies were grouped according to the continent. The results are presented in the narrative text.

This study was based exclusively on published literature and did not require ethical approval.

## RESULTS

**FIGURE 1:**
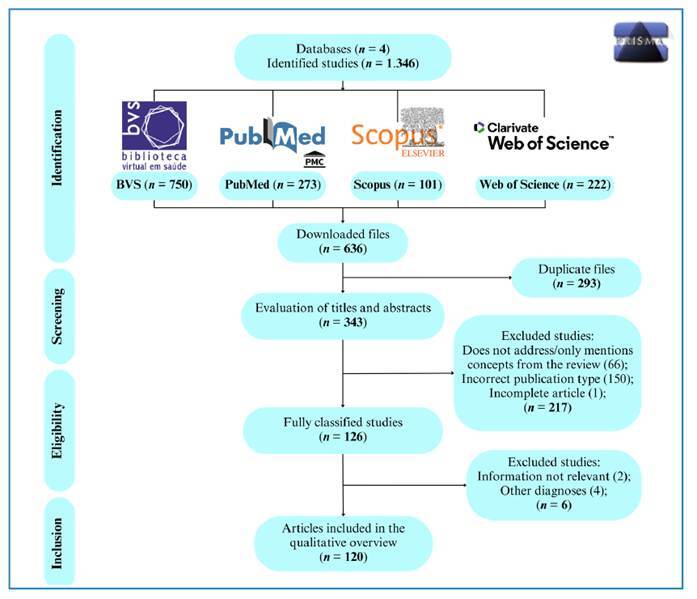
PRISMA flowchart of the selection and screening process.

The electronic search identified 1,346 records, of which 636 were downloaded from the databases. After removing duplicate files (293), the titles and abstracts of the remaining files (344) were assessed for eligibility. In total, 127 articles were selected for full-text reading and reference screening, of which 120 were included in this review (Figure 1). The main limitation was that some clinical data from the included studies were incomplete.

### Worldwide Epidemiological Characteristics

**FIGURE 2:**
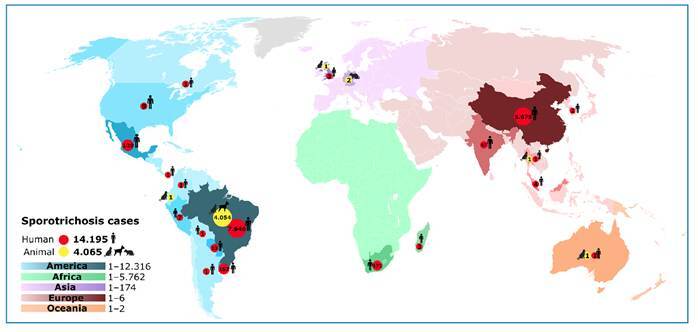
Geographic distribution of reported cases for Sporotrichosis (2018-2022).

A total of 120 publications reported 18,251 cases of *Sporothrix* infections worldwide between 2018 and 2022. Of the 14,187 human cases with available sex data, 2,737 involved male patients and 3,973 involved female patients. The Americas accounted for 67.48% (*n* = 12,315) of all cases^9^ ^,^ ^12^ ^-^ ^99^, followed by Asia (5,754 cases, 40.56%)^100^ ^-^ ^118^. Brazil and China recorded the highest burden with Brazil reporting 7,945 human and 4,058 animal cases, and China reporting 5,679 human cases (Figure 2). Animal infections totaled 4,064 cases-3,703 in felines, 359 in canines, and 2 in marsupials-most reported in Brazil (*n* = 4,058).

### Sporotrichosis in the Americas

Ninety-two publications reported 12,315 cases of sporotrichosis in the Americas^9^ ^,^ ^12^ ^-^ ^99^. In Canada, only one case has been reported in Ontario was described^92^. Nine cases have been documented in the United States: three in California, two in Texas, and one each in North Carolina, Kansas, Oklahoma, and Florida^17^ ^,^ ^42^ ^,^ ^40^ ^,^ ^44^ ^,^ ^62^ ^,^ ^73^ ^,^ ^89^ ^,^ ^93^ ^,^ ^98^. Mexico accounted for 120 notified cases, led by Guerrero (76 cases), with one case each in Mexico City, Chihuahua, Ixtapaluca, and Jalisco; the remaining 39 cases lacked specific regional data^15^ ^,^ ^32^ ^,^ ^38^ ^,^ ^67^ ^,^ ^73^ ^,^ ^84^. La Chorrera in Panama was the sole Central American locality with a reported clinical case^83^. In South America, Brazil was dominated by 12,003 cases, spanning ten states-most notably Rio de Janeiro (6,539 cases) and Minas Gerais (626 cases). Other affected locations included Belo Horizonte, the Federal District, Espírito Santo, Pernambuco, Rio Grande do Norte, Rio Grande do Sul, São Paulo, and Santa Catarina (totaling 780 cases)^9^ ^,^ ^14^ ^,^ ^16^ ^,^ ^18^ ^-^ ^24^ ^,^ ^26^ ^-^ ^27^ ^,^ ^29^ ^-^ ^31^ ^,^ ^33^ ^-^ ^37^ ^,^ ^39^ ^-^ ^41^ ^,^ ^43^ ^,^ ^45^ ^-^ ^51^ ^,^ ^53^ ^,^ ^55^ ^,^ ^57^ ^-^ ^66^ ^,^ ^68^ ^-^ ^71^ ^,^ ^76^ ^-^ ^79^ ^,^ ^81^ ^-^ ^82^ ^,^ ^86^ ^-^ ^88^ ^,^ ^90^ ^-^ ^91^ ^,^ ^94^ ^-^ ^97^. Other South American countries reporting sporotrichosis included Argentina, Bolivia, Colombia, and Ecuador (one case each), Paraguay (13 cases), Peru (7 cases), and Uruguay (157 cases)^12^ ^-^ ^13^ ^,^ ^25^ ^,^ ^28^ ^,^ ^44^ ^,^ ^54^ ^,^ ^75^ ^,^ ^80^ ^,^ ^98^.

### Sporotrichosis in Asia

Nineteen publications reported 5,762 cases of sporotrichosis in Asia^100^ ^-^ ^118^. China accounted for the vast majority of cases, with 5,675 human cases, primarily from Jilin Province. Single cases have also been documented in Chengdu, Shandong, Shiyan, and Tianjin^101^ ^-^ ^102^ ^,^ ^107^ ^-^ ^108^ ^,^ ^113^ ^,^ ^116^ ^-^ ^117^. In South Korea, ten cases have been recorded in Daegu^104^ ^-^ ^105^. India reported 63 clinical cases in Chandigarh, three in Manipur, and one in Himachal Pradesh^114^ ^-^ ^115^ ^,^ ^118^. In Malaysia, Kelantan had four cases and Kota Bharu had one case^103^ ^,^ ^106^ ^-^ ^107^ ^,^ ^109^ ^,^ ^111^ ^-^ ^112^. Thailand’s only reports are from Bangkok, with one human and one animal case^100^ ^,^ ^110^.

### Sporotrichosis in Africa, Europe, and Oceania

Notifications from Africa, Europe, and Oceania were less frequent. South Africa reported 171 cases, and Madagascar reported three cases. The United Kingdom has documented three human and three animal cases, while Australia has recorded one human and one animal case^1^ ^,^ ^119^ ^,^ ^120^ ^-^ ^127^.

The species-level identifications obtained by molecular assays were as follows: *S. brasiliensis* (1,156 isolates), the pathogenic clade (“*S. schenckii* complex,” 791 isolates), and *S. schenckii* sensu stricto (100 isolates). The less frequently encountered species included *S. globosa* (13 isolates), *S. humicola* (3 isolates), and *S. chilensis* (1 isolate). Additionally, 775 samples were identified only at the genus level (*Sporothrix* sp.) and 12,371 isolates remained unidentified.

Among the 6,772 cases with a defined clinical form, 99.4% (n = 6,734) were classified as cutaneous. Of these, the fixed cutaneous form predominated (n = 4,706), followed by the lymphocutaneous (n = 1,956) and disseminated cutaneous (n = 72) presentations. Thirty-seven cases were categorized as extracutaneous sporotrichosis, while 197 lacked sufficient data for clinical classification.

Culture-based mycological methods were the most commonly employed diagnostic approaches, reported in 74 studies, followed by genotypic identification polymerase chain reaction (PCR), as cited in 45 publications. Other techniques such as direct microscopic examination (DME), matrix-assisted laser desorption/ionization time-of-flight mass spectrometry (MALDI-TOF MS), enzyme-linked immunosorbent assay (ELISA), and sputum/bronchoalveolar lavage (BAL) analysis are rarely used in routine diagnostics^24^ ^-^ ^60^ ^,^ ^110^ ^-^ ^119^.

Treatment regimens varied; itraconazole (ITZ) was the most widely applied therapy, used in 2,022 cases, either as monotherapy or in moderate-to-severe infections, in combination with other antifungals given simultaneously or sequentially. Saturated potassium iodide (KI) solution was the next most prevalent treatment (956 cases), followed by amphotericin B (AmB; 695 cases) and terbinafine (TBF; 197 cases). Less common therapies, including posaconazole, fluconazole, and ketoconazole, and adjunctive approaches, such as thermotherapy and cryosurgery, were reserved for cases of drug intolerance, contraindication, or suboptimal response^24^ ^,^ ^32^ ^,^ ^38^ ^,^ ^39^ ^,^ ^47^ ^-^ ^48^ ^,^ ^60^ ^,^ ^66^ ^,^ ^90^ ^,^ ^114^ ^,^ ^124^ ^,^ ^126^.

## DISCUSSION

Data from 2018 to 2022 confirmed the global presence of sporotrichosis, with the highest incidence in South America (Brazil), Asia (China), and Africa (South Africa). This distribution reflects concentrations in tropical and subtropical climates, which favor the growth of *Sporothrix* species in soil, organic matter, and animal hosts-particularly cats^10^. The observed geographic expansion, such as the recent detection of *S. brasiliensis* in the United Kingdom, suggests that climatic factors and pet movement may extend the risk zones^128^.

In terms of animal sporotrichosis, Brazil led to the most reported cases during the study period. Animal cases remain rare in Europe^121^ and Africa^127^ although human and animal infections have become increasingly common. In Australia, sporadic human cases occur only in the few hundreds^2^.

For many years, *S. schenckii* was considered the sole etiological agent of sporotrichosis. Advances in *Sporothrix* taxonomy have revealed extensive genetic diversity. The 53 species described fall into two major clades: the clinical/pathogenic clade, which includes *S. brasiliensis*, *S. schenckii*, *S. globosa*, and *S. luriei*, species frequently isolated from human and animal cases, and the environmental clade, which comprises five groups associated with substrates ranging from soil and decaying organic matter to insects and plants^5^.

The predominance of *S. brasiliensis* and the high number of feline cases underscores the role of cats as zoonotic amplifiers^129^, corroborating the hyperendemic outbreaks recorded in Brazil’s southeastern region^119^. Previously, human sporotrichosis has been reported in 25 of Brazil’s 26 states^2^ ^,^ ^41^ ^,^ ^130^ ^-^ ^131^.

Conversely, reports referencing the pathogenic “*S. schenckii* sensu lato” complex (791 isolates) have been published, highlighting the need for taxonomic updates and the systematic use of PCR to accurately define species distribution^5^ ^,^ ^132^.

Among the environmental clades, *S. chilensis* and *S. humicola* were the only species identified in the reviewed studies. Although rare among mammalian pathogens^123^, these species can occasionally cause infections following direct contact with contaminated materials^6^.

A clinical analysis of 6,772 cases with a defined form showed that fixed cutaneous and lymphocutaneous presentations accounted for over 99% of diagnoses. The predominance of these forms reflects both the classic pathogenesis, subcutaneous invasion with lymphatic involvement^7^ ^,^ ^23^ ^,^ ^133^ ^,^ ^134^- and limited access to confirmatory testing in rural areas, where culture remains the gold standard^135^ despite being slow and prone to contamination.

Lymphocutaneous and fixed cutaneous forms are commonly reported worldwide^2^ ^,^ ^5^ ^-^ ^10^; the lymphocutaneous type is the most frequent, as it primarily affects the lymphatic vessels and subcutaneous tissue of the skin, producing ulcers and impairing lymphatic function^134^. Conversely, the fixed cutaneous form, which is typically localized in the skin, was the most prominent in our data, possibly indicating robust host immune responses that prevent dissemination to other organs or tissues^5^. Extracutaneous (disseminated/systemic) sporotrichosis can result in respiratory and pulmonary complications, osteomyelitis, arthritis, meningitis, and other rare manifestations, particularly in immunosuppressed individuals^133^, which is consistent with the low frequency reported in this review.

In this context, genotypic identification methods have gained increasing research interest, with various genetic and molecular sequencing techniques, such as PCR, being employed for diagnostic testing^2^ ^,^ ^5^.

It is important to note that most of the reviewed studies recommend a thorough differential diagnosis, as the clinical signs of sporotrichosis are not specific and may be observed in other conditions such as actinomycosis, cryptococcosis, mycobacteriosis, nocardiosis, pyoderma, syphilis, neoplasms, lupus, pemphigus vulgaris, cutaneous leishmaniasis, granuloma annulare, osteomyelitis, rheumatoid and pulmonary tuberculosis, cat-scratch disease, among others^136^.

Itraconazole (ITZ) is the first-line treatment for sporotrichosis owing to its 90-100% efficacy, safety, and convenient dosing, even at low doses^136^. Therapeutic doses range from 100 to 400 mg/day, depending on the disease severity and host immune status. Despite its effectiveness, ITZ can cause significant side effects and interact with over 200 other medications, leading to adverse events (e.g., headache, gastrointestinal disturbances, hepatotoxicity, teratogenicity, and embryotoxicity) or therapeutic failure^137^.

Treatment selection depends on the clinical form, host immune status, and infection with the *Sporothrix* species. The most virulent species (*S. brasiliensis*, *S. schenckii*, and *S. globosa*) exhibit variable antifungal susceptibility profiles, which result in heterogeneous treatment responses^138^.

Although potassium iodide (KI) has been used since 1903 for sporotrichosis treatment^136^, its fungicidal mechanism remains unclear. Brilhante et al.^139^ demonstrated that KI inhibited biofilm formation in some *Sporothrix* species. Its frequent use in our review (956 cases) is likely due to its low cost and rapid clinical response^140^, and is often administered alongside ITZ for cutaneous forms in humans and animals^3^ ^,^ ^136^.

Terbinafine (TBF) is recommended for cutaneous sporotrichosis in humans when ITZ or KI is not tolerated or is contraindicated^141^. Amphotericin B (AmB) is reserved for life-threatening cases, but is not recommended for cutaneous or lymphocutaneous diseases because of its high toxicity and intravenous administration challenges^142^.

While ITZ, KI, and AmB remain cornerstone therapies, their toxicity and drug-drug interactions underscore the need for safer alternatives, especially in patients with comorbidities^137^ ^-^ ^138^. Research on novel antifungals and adjunctive therapies (e.g., cryotherapy and thermotherapy) may address these gaps.

Finally, our findings support a One Health approach that integrates human, veterinary, and environmental surveillance^2^ ^,^ ^9^ ^-^ ^10^. Practical measures emerging from our epidemiological data include the following.

- Use of appropriate personal protective equipment (PPE) in rural settings and during clean-up of yards or organic debris
- Responsible management of infected animals and safe disposal of carcasses;
- Ongoing education for animal owners and communities is needed to raise awareness of sporotrichosis and its impacts on human, animal, and environment^3^ ^-^ ^4^ ^,^ ^143^.

Future studies should focus on antifungal resistance and climate-driven predictive models to guide interventions in high-risk regions.

## CONCLUSION

The current situation demonstrates the emergence of sporotrichosis as a cosmopolitan mycosis, with 18,251 cases reported worldwide. It is likely that this number was underestimated, mainly because the disease is mandatorily notifiable in only a few countries. This review provides supporting data on the worldwide epidemiological characteristics of sporotrichosis. Sporotrichosis is prevalent in tropical zones, especially Brazil, where the highest numbers of human and feline cases have been recorded. The clinical manifestation of sporotrichosis is, for the most part, the cutaneous fixed type. Fungal culture is the most common diagnostic method for identifying the etiological agent; however, molecular tools are needed to identify and monitor *Sporothrix* spp., especially *S. brasiliensis*, *S. schenckii*, and species of the pathogenic clade, which are frequently reported in South America (Brazil, Mexico, and Uruguay) and Asia (China and India). Prevention and control measures require interdisciplinary teams to develop effective and sustainable responses in collaboration with managers, public health authorities, physicians, biologists, veterinarians, community agents, volunteers, and other allies to implement health promotion, reduce sporotrichosis cases, and face this threat to global health.

## Data Availability

Research data is only available upon request.
